# Allelic variation at the vernalization and photoperiod sensitivity loci in Chinese winter wheat cultivars (*Triticum aestivum* L.)

**DOI:** 10.3389/fpls.2015.00470

**Published:** 2015-07-01

**Authors:** Xiangfen Zhang, Manxia Gao, Shasha Wang, Feng Chen, Dangqun Cui

**Affiliations:** ^1^Collaborative Innovation Center of Henan Grain CropsZhengzhou, China; ^2^National Key Laboratory of Wheat and Maize Crop ScienceZhengzhou, China; ^3^Agronomy College, Henan Agricultural UniversityZhengzhou, China

**Keywords:** bread wheat, vernalization genes, photoperiod genes, copy number variations, allelic variation

## Abstract

A total of 205 wheat cultivars from the Yellow and Huai valley of China were used to identify allelic variations of vernalization and photoperiod response genes, as well as the copy number variations (CNVs) of *Ppd-B1* and *Vrn-A1* genes. A novel *Vrn-D1* allele with 174-bp insertion in the promoter region of the recessive allele *vrn-D1* was discovered in three Chinese wheat cultivars and designated as *Vrn-D1c*. Quantitative real-time polymerase chain reaction showed that cultivars with the *Vrn-D1c* allele exhibited significantly higher expression of the *Vrn-D1* gene than that in cultivars with the recessive allele *vrn-D1*, indicating that the 174-bp insertion of *Vrn-D1c* contributed to the increase in *Vrn-D1* gene expression and caused early heading and flowering. The five new *cis*-elements (Box II-like, 3-AF1 binding site, TC-rich repeats, Box-W1 and CAT-box) in the 174-bp insertion possibly promoted the basal activity level of *Vrn-D1* gene. Two new polymorphism combinations of photoperiod genes were identified and designated as *Ppd-D1_Hapl-IX* and *Ppd-D1_Hapl-X*. Association of the CNV of *Ppd-B1* gene with the heading and flowering days showed that the cultivars with *Ppd-B1_Hapl-VI* demonstrated the earliest heading and flowering times, and those with *Ppd-B1_Hapl-IV* presented the latest heading and flowering times in three cropping seasons. Distribution of the vernalization and photoperiod response genes indicated that all recessive alleles at the four vernalization response loci, *Ppd-B1_Hapl-I* at *Ppd-B1* locus, and *Ppd-D1_Hapl-I* at the *Ppd-D1* locus were predominant in Chinese winter wheat cultivars. This study can provide useful information for wheat breeding programs to screen wheat cultivars with relatively superior adaptability and maturity.

## Introduction

Heading and flowering times of bread wheat, mainly modulated by vernalization and photoperiod genes, are important factors that determine the adaptation of wheat plants to different environmental conditions and influence the growth and productivity of wheat (Law and Worland, 1997). Vernalization is the acquisition of a plant's ability to flower in the spring by exposure to the prolonged cold of winter, or by an artificial equivalent. Photoperiod insensitivity is widespread in the world's wheat varieties and predominates in regions where spring wheat is grown as a crop over the winter period and where autumn-sown winter wheat needs to mature in the following year before the onset of high summer temperatures.

To date, three fundamental vernalization response genes (*Vrn-1, Vrn-2*, and *Vrn-3*) have been discovered in polyploid wheat and barley. *Vrn-1* genes, encompassing *Vrn-A1, Vrn-B1*, and *Vrn-D1* genes on the long arms of chromosome 5 (Law et al., 1975; Galiba et al., 1995; Dubcovsky et al., 1998; Barrett et al., 2002; Iwaki et al., 2002), are upregulated by vernalization treatment, and their overexpression can accelerate flowering and maturity of wheat (Yan et al., 2003). The *Vrn-2* gene, a dominant repressor of flowering, is downregulated by vernalization treatment. Loss of functional mutation of *Vrn-2* resulted in the spring growth of bread wheat to head and flower under non-vernalization treatment. The *Vrn-3* gene is an ortholog of the *Arabidopsis FT* (flowering time) gene and is upregulated by vernalization treatment (Yan et al., 2006). A molecular model explaining the *Vrn-1/Vrn-2/Vrn-3* epistatic interaction in winter wheat was proposed by Yan et al. (2003, 2004a,b) and Chen and Dubcovsky (2012). Moreover, different vernalization alleles at *Vrn-1* and *Vrn-3* loci were discovered. The *Vrn-A1a* allele exhibits an insertion of a foldback repetitive element, as well as a duplicated region in the promoter, causing a strong effect on vernalization response and resulting in the complete elimination of the vernalization requirement. The *Vrn-A1b, Vrn-A1c, Vrn-A1d*, and *Vrn-A1e* have been subsequently described in polyploid wheat (Yan et al., 2004a; Fu et al., 2005). The different *Vrn-B1* alleles (*Vrn-B1a, Vrn-B1b*, and *Vrn-B1c*) and *Vrn-D1* alleles (*Vrn-D1a, Vrn-D1b*, and *Vrn-D1s*) are mostly results of insertion and/or deletion in the intron1 region (Yan et al., 2004a; Fu et al., 2005; Santra et al., 2009; Milec et al., 2012; Shcherban et al., 2012; Zhang et al., 2012; Muterko et al., 2014). Plants homozygous for the *Vrn-D1b* allele headed 32 days later than plants homozygous for the *Vrn-D1a* allele, and that the*Vrn-D1b* gene is associated with facultative growth habit (Zhang et al., 2012). The *Vrn-B3a* allele is a 5300-bp insertion in the promoter region, and *Vrn-B3b* and *Vrn-B3c* alleles were recently discovered in bread wheat (Yan et al., 2006; Chen et al., 2013a). Chen et al. (2013a) indicated that *Vrn-B3b* significantly reduced the expression level of the gene and caused later heading and flowering compared with the *vrn-B3* gene. Additionally, copy number variation (CNV) slightly influenced the vernalization gene expression and showed little effect on the phenotype. The predominant result is a C/T double peak in sequence trace files in exon 4 on *Vrn-A1*, and plants with an increased CNV showed an increased requirement for vernalization, thereby requiring longer cold treatment to potentiate flowering (Diaz et al., 2012).

Photoperiod response is another important factor influencing the start and length of the flowering period. The important photoperiod response genes are *Ppd-A1, Ppd-B1*, and *Ppd-D1*, which are located in 2A, 2B, and 2D of the *Ppd-1* loci, respectively (Worland et al., 1998; Shitsukawa et al., 2007). Genetic studies showed that the most effective photoperiod insensitivity gene is the *Ppd-D1* gene, followed by *Ppd-B1* and *Ppd-A1*. Beales et al. (2007) detected a 2089-bp deletion in the promoter region of the *Ppd-D1a* gene, which is the photoperiod-insensitive allele. The second polymorphism was a mariner-like transposable element (TE) present in intron 1; the third was a 5-bp deletion in exon 7; the fourth was a SNP (A/G) in exon 7; the fifth was a 16-bp deletion including the last two bases of the CCT domain in exon 8. Guo et al. (2010) described these alleles to six polymorphisms, involving one photoperiod-insensitive haplotype and five photoperiod-sensitive haplotypes in *Ppd-D1*. Chen et al. (2013a) also identified two new polymorphism combinations in *Ppd-D1* of common wheat. Diaz et al. (2012) reported that the *Ppd-B1* gene contained three CNVs, namely, truncated “Chinese Spring” *Ppd-B1* allele, intact “Chinese Spring” *Ppd-B1* allele, and intact “Sonora64” *Ppd-B1* allele, and proved that the alleles with higher copy number of *Ppd-B1* confer an early flowering day neutral phenotype.

China is the largest wheat consumer and producer worldwide. Winter wheat occupies more than 85% of the total area and production of Chinese wheat. China has 10 major agro-ecological zones that are further divided into 26 subzones (Zhuang, 2003). Among the agro-ecological zones, the Yellow and Huai wheat production region is the most important and largest wheat production zone, with 60–70% of both total harvested area and total wheat production. In a previous study (Chen et al., 2013a), we characterized the vernalization and photoperiod response genes in currently popular cultivars and landraces from the Yellow and Huai wheat region. In the present research, we further identified the molecular characterization of the vernalization and photoperiod response genes in backbone parents in the wheat breeding program of this wheat region and found a new *Vrn-D1* allele, a *Vrn-D1* null allele, two new polymorphism combinations of photoperiod genes, and several CNVs. Our results provide useful information for wheat breeding programs to screen relatively superior wheat germplasms in view of their adaptability to diverse agronomic environments.

## Materials and methods

### Plant materials

A total of 205 winter wheat cultivars and advanced lines were planted in 2011–2012, 2012–2013, and 2013–2014 cropping seasons, respectively, at the Zhengzhou Scientific Research and Education Center of Henan Agricultural University (N34.9; E113.6) under local management practices. All surveyed cultivars were vernalized through winter with an average temperature of 1.3°C (December, January, and February) in 2012, 2013, and 2014. The wheat germplasms used were important landrace, historical, and introduced cultivars in China, especially in the Yellow and Huai wheat region. Different from the materials we previously used (Chen et al., 2013a), these germplasms were mainly used as backbone parents and had played important roles in wheat breeding programs in China. The field experiment was conducted using a completely randomized design. Each plot contained four 200 cm-long rows with 23 cm between neighboring rows and 10 cm between neighboring plants. All surveyed cultivars grew very well with the supporting net without lodging. The heading and flowering times of each cultivar were investigated in April 2012, April 2013, and April 2014, and their heading and flowering days were calculated from the sowing day to the heading and flowering days.

The wheat plants of four Chinese cultivars (Yanzhan 4110, Jinmai 50, Lumai 19, and Yunong 876) were grown in a greenhouse under 16 h light at 25°C–28°C (day) and 8 h dark at 20°C–22°C (night) to investigate the heading and flowering days under the condition of non-vernalization treatment.

### Polymerase chain reaction (PCR) parameters and DNA sequencing

The genomic DNA of each cultivar surveyed was individually extracted from three pulverized kernels as described by Chen et al. (2011). The PCR reactions and programs were performed according to Chen et al. (2013a) (detailed annealing temperatures in Supplemental Table 1). PCR products were separated on a 1.5–2.5% agarose gel stained with ethidium bromide and visualized with UV light or on a 6% polyacrylamide gel and resolved by silver staining.

After purification using Quick DNA Extraction Kit (Takara, http://www.takara.com.cn/), targeted PCR products were ligated into pGEM-T Easy vector and transformed into competent cells of an *Escherichia coli DH-5α* strain. Plasmids with targeted fragments, detected by colony PCR, were extracted by Plasmid Rapid Isolation Kit (Biodev-tech Company, http://biodev.technew.cn/). Five subclones for each PCR product were sequenced from both strands by SinoGenoMax Co., Ltd. (http://www.sinogenomax.com/). Analysis and multiple alignments of sequences were performed by DNAMAN Version 6.0, and graphic data were analyzed to check the reliability of the sequencing results by using Chromas Version 1.4.5 and FinchTV version 1.4.0.

### Real-time quantitative reverse transcription PCR

Total RNAs of the four Chinese cultivars (Yanzhan 4110, Jinmai 50, Lumai 19, and Yunong 876) with different *Vrn-D1* alleles were extracted from 2-month-old seedlings for real-time quantitative reverse transcription PCR (qRT-PCR) as described by Chen et al. (2013b). The primer set Vrn-P17F/R (Supplemental Table 1) was designed by Software Primer Premier 5.0 for qRT-PCR amplification. Amplification with *β*-actin primers was used as internal control to normalize all the data. Relative quantification method (2^−ΔΔ^CT) was used to evaluate the quantitative variation among the three replicates.

## Results

### Discovery of a novel dominant *Vrn-D1* allele in chinese winter wheat

**Figure 1:**
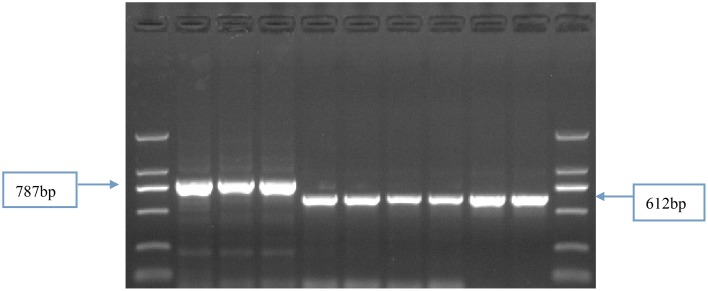
**Identification of vernalization response alleles by Vrn-P10F/R markers in the Chinese winter wheat cultivars**. Primer set Vrn-10F/R for identification of *Vrn-D1c* from *Vrn-D1* promoter with 787-bp fragment. From L to R: DNA ladder DL2000. 876 for three lanes, Shuangji 2, Shuangji 4, Jingshuang 16, Lumai 14, Ji 87 guan 739. DL2000.

**Figure 2:**
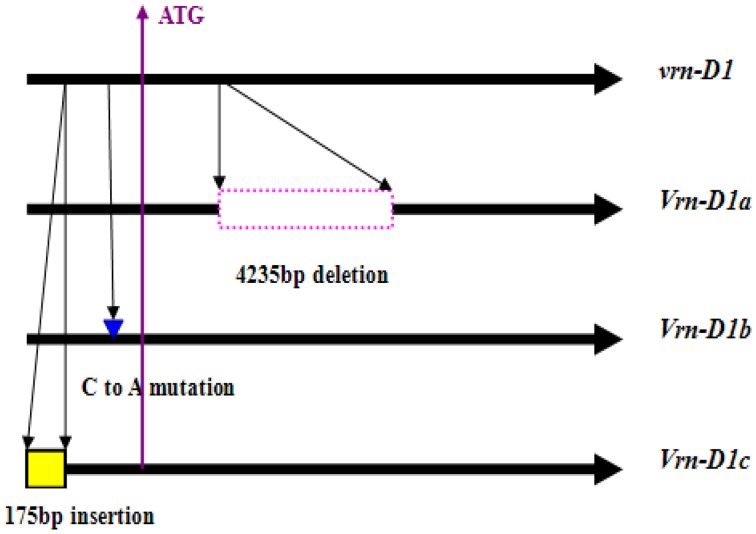
**Schematic of *Vrn-D1* alleles identified in the Chinese bread wheat cultivars**.

Identification of the 205 Chinese winter wheat cultivars by four primer sets (Vrn-P8F/R, Vrn-P9F/R, Vrn-P10F/R, and Vrn-P11F/R; Supplemental Table 1) indicated that 119 cultivars (58.0%) contained the recessive allele *vrn-D1*, and 59 (28.8%) and 24 (11.7%) cultivars contained the dominant alleles *Vrn-D1a* and *Vrn-D1b*, respectively. However, the remaining three cultivars showed an approximately 800-bp fragment when amplified with the primer set Vrn-P10F/R. The presence of this fragment indicated a nearly 200-bp insertion in these three cultivars compared with the recessive *vrn-D1* allele (Figure 1). The sequencing results indicated that a 174-bp fragment was inserted into the 5′-UTR at -601-bp (relative to ATG) of the *vrn-D1* gene (Table 1 and Figure 2). This new *Vrn-D1* allele with 174-bp insertion was designated as *Vrn-D1c* allele (submitted to NCBI No.: KP721800) in accordance with the nomenclature of vernalization response genes by Fu et al. (2005), Yan et al. (2004a, 2006) and Chen et al. (2013a).

**Table 1:** **Molecular characterization of vernalization response alleles in polyploid wheat**.

**Locus**	**Allele**	**NCBI No**.	**Molecular characterization**	**References**
*Vrn-A1*	*vrn-A1*	AY747600	–	Fu et al., 2005
	*Vrn-A1a*	AY616458, AY616459	231-bp and 140-bp insertions at −439 and −348 bp, respectively	Yan et al., 2004a
	*Vrn-A1b*	AY616461	20-bp deletion at −157 bp	Yan et al., 2004a
	*Vrn-A1c*	AY747599	5504-bp deletion at +1349 bp	Fu et al., 2005
	*Vrn-A1d*	AY616462	32-bp deletion at −214 bp	Yan et al., 2004a
	*Vrn-A1e*	AY616463	54-bp deletion at −220 bp	Yan et al., 2004a
*Vrn-B1*	*vrn-B1*	AY747604	–	Fu et al., 2005
	*Vrn-B1a*	AY747603	6850-bp deletion at +836 bp	Fu et al., 2005
	*Vrn-B1b*	FJ766015	6850-bp deletion at +836 bp and 37-bp deletion at +7992 bp	Santra et al., 2009
	*Vrn-B1c*	HQ593668, HQ130482	817-bp deletion and 0.4-kb duplication at +798 bp	Milec et al., 2012; Shcherban et al., 2012
*Vrn-B3*	*vrn-B3*	DQ890162	–	Yan et al., 2006
	*Vrn-B3a*	DQ890165	5300-bp insertion at −592 bp	Yan et al., 2006
	*Vrn-B3b*	JN627519	890-bp insertion at −429 bp	Chen et al., 2013a
	*Vrn-B3c*	JQ082311	5300-bp insertion at −592 bp but 20-bp and 4-bp deletions at −3543 and −3591 bp	Chen et al., 2013a
*Vrn-D1*	*vrn-D1*	AY747606	–	Fu et al., 2005
	*Vrn-D1a*	AY747597	4235-bp deletion at +810 bp	Fu et al., 2005
	*Vrn-D1b*		C mutation to A at −161 bp	Zhang et al., 2012
	*Vrn-D1s*	KF800714	844-bp deletion at +1044 bp	Muterko et al., 2014
	*Vrn-D1c*	KP721800	174-bp deletion at −601 bp	In this paper

### Expression level of the *Vrn-D1* gene associated with DH and DF in winter wheat

**Figure 3:**
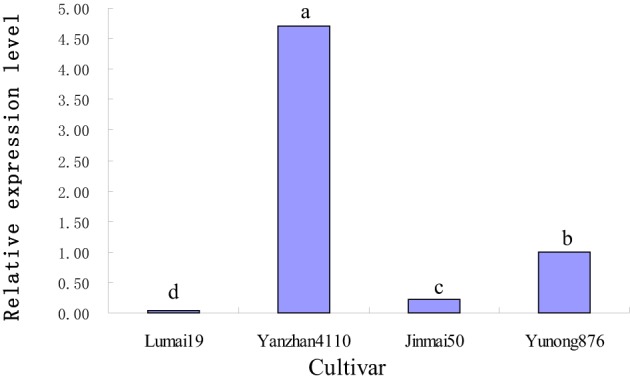
**Comparison of relative expression levels of four cultivars with different *Vrn-D1* alleles by real-time PCR**.

**Figure 4:**
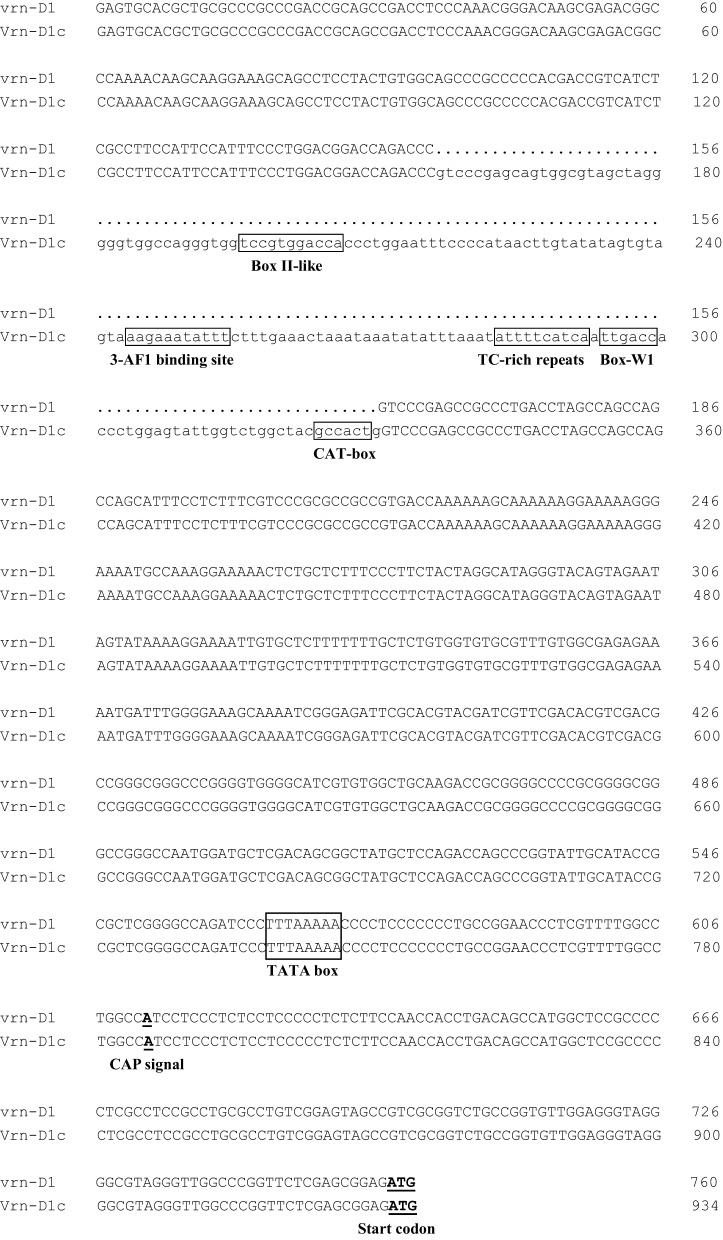
**Sequence alignment in *Vrn-D1* promoter region between the recessive *vrn-D1* allele and the dominant *Vrn-D1c* allele**. The ATG start codon and the putative CAP signal for transcription initiation are indicated in bold and underlined. Five predicted *cis*-elements (Box II-like, 3-AF1 binding site, TC-rich repeats, Box-W1, and CAT-box) and the TATA box are indicated in a rectangle.

Four cultivars, namely, Lumai19 (with the recessive allele *vrn-D1*), Yanzhan 4110 (with *Vrn-D1a* allele), Jinmai 50 (with *Vrn-D1b* allele), and Yunong 876 (with *Vrn-D1c* allele), were selected to analyze the expression levels of different *Vrn-D1* alleles by qRT-PCR (Figure 3) owing to their same *Vrn-A1, Vrn-B1, Vrn-B3*, and *Ppd-D1* alleles. The qRT-PCR results indicated that Lumai 19 exhibited the lowest expression level of *Vrn-D1* gene. Jinmai 50 demonstrated a significantly higher expression than that of Lumai 19, suggesting that a single point mutation significantly increased the *Vrn-D1* gene expression (C-to-A mutation in Jinmai 50 allele in Figure 2). *Vrn-D1* gene in Yunong 876 showed significantly higher expression levels than those of Lumai19 and Jinmai 50, suggesting that the 174-bp insertion possibly contributed to the increased expression. Among the four cultivars, Yanzhan 4110 showed the highest expression level of the *Vrn-D1* gene. The prediction of *cis* elements in promoters of *Vrn-D1* genes by software PlantCARE (http://bioinformatics.psb.ugent.be/webtools/plantcare/html/) indicated that five new *cis*-elements (Box II-like, 3-AF1 binding site, TC-rich repeats, Box-W1 and CAT-box) were generated in the 174-bp insertion of *Vrn-D1c* gene promoter (Figure 4). Presence of these five *cis* elements possibly resulted in the significantly increased basal activity level of *Vrn-D1c* when compared with the recessive *vrn-D1* allele.

Phenotype investigation of these four cultivars under vernalization treatment showed that the heading and flowering days of the cultivar Lumai 19 with recessive allele *vrn-D1* were 195 and 200 days, respectively. Jinmai 50 (194 DH, 199 DF) with the *Vrn-D1b* allele headed and flowered 1 days ahead of Lumai 19. Yanzhan 4110 (192 DH, 196 DF) with the *Vrn-D1a* allele headed 3 days ahead and flowered 4 days ahead of Lumai 19. Yunong 876 (193 DH, 200 DF) with the *Vrn-D1c* allele headed 2 days ahead of Lumai 19 but flowered on the same day. These results suggested that the 174-bp insertion of the *Vrn-D1c* allele possibly prolonged the heading to flowering days of bread wheat under vernalization treatment.

Moreover, phenotype investigation of the four cultivars under non-vernalization treatment showed that the heading and flowering days of the cultivar Lumai 19 were 120 and 122 days, respectively. Jinmai 50 (111 DH, 114 DF) headed 9 days ahead and flowered 8 days ahead of Jinmai 50. Yanzhan 4110 (61 DH, 72 DF) headed 59 days ahead and flowered 50 days ahead of Lumai 19. Yunong 876 (76 DH, 80DF) with the *Vrn-D1c* allele headed 44 days ahead and flowered 46 days ahead of Lumai 19. These results suggested that the expression of the *Vrn-D1* genes promotes early heading and flowering under non-vernalization treatment.

### Distribution of vernalization response alleles in chinese winter wheat cultivars

Identification with four allele-specific primer sets (Vrn-P1F/R, Vrn-P2F/R, Vrn-P3F/R, and Vrn-P4F/R in Supplemental Table 1) showed that 198 out of 205 (96.6%) winter wheat cultivars contained the recessive *vrn-A1* allele, and five and two wheat cultivars contained *Vrn-A1a* and *Vrn-A1b* alleles in the *Vrn-A1* locus (Table 2), respectively. Identification with three primer sets (Vrn-P5F/R, Vrn-P6F/R, and Vrn-P7F/R) indicated that 172 out of 205 (83.90%) winter wheat cultivars contained the *vrn-B1*, and 24 and 9 cultivars contained *Vrn-B1a* and *Vrn-B1b* alleles in the *Vrn-B1* locus (Table 2), respectively. Identification with four primer sets (Vrn-P8F/R, Vrn-P9F/R, Vrn-P10F/R, and Vrn-P11F/R) showed that 118 out of 205 (57.56%) winter cultivars contained the *vrn-D1*, and 60 (29.27%) and 23 (11.22%) out of the remaining 87 cultivars contained *Vrn-D1a* and *Vrn-D1b* alleles in the *Vrn-D1* locus (Table 2), respectively. Three cultivars exhibited the *Vrn-D1c* allele as mentioned above. Surprisingly, cultivar Pincun 16 did not yield any PCR amplification result when amplified with primer sets Vrn-P8F/R, Vrn-P9F/R, Vrn-P10F/R, and Vrn-P11F/R, indicating the lack of *Vrn-D1* gene. Identification results with the four primer sets (Vrn-P12F/R, Vrn-P13F/R, Vrn-P14F/R, and Vrn-P15F/R) showed that Yanda 1817, which has been widely used as a parent in Chinese wheat breeding programs, contained a rare allele *Vrn-B3b* (recently reported by Chen et al., 2013a), and three cultivars contained the dominant allele *Vrn-B3a*. All the remaining cultivars (98.05%) contained the recessive allele *vrn-B3* in the *Vrn-B3* locus. These results suggested that the recessive allele *vrn-A1, vrn-B1, vrn-D1*, and *vrn-B3* were predominant in the *Vrn-A1, Vrn-B1, Vrn-D1*, and *Vrn-B3* loci. But the dominant *Vrn-D1a* and *Vrn-D1b* alleles were also prevalent genotypes (Table 2). These results were consistent with the previous findings of Zhang et al. (2008) and Chen et al. (2013a).

**Table 2:** **The distribution of vernalization and *Ppd-D1* genes in the Chinese bread wheat cultivars surveyed**.

**Locus**	**Allele**	**Number of cultivars with an allele**	**Frenquency (%)**	**Typical cultivars**
*Vrn-A1*	*vrn-A1*	198	96.6	Abbondanza; Orofen; Nonglin 10
	*Vrn-A1a*	5	2.44	SW604; SW601; SW618
	*Vrn-A1b*	2	0.98	605; Triumph
*Vrn-B1*	*vrn-B1*	172	83.9	Nonglin 10; Shuiyuan 86; Lovin 10
	*Vrn-B1a*	24	11.7	Lankao 4; Orofen; SW604
	*Vrn-B1b*	9	4.39	Beijing 841; Xinong 164; Yangmai 4
*Vrn-D1*	*vrn-D1*	120	58.5	Jingshuang 16; Beijing 6; Zhongnong 28
	*Vrn-D1a*	59	28.8	Yanzhan 1; XJ1; Mianyang 8640
	*Vrn-D1b*	23	11.2	Jinmai 50; Zhengmai 9201; Fengyou 8
	*Vrn-D1c*	3	1.46	Shannong 413863; Xinmai 18^*^; Yunong 876
*Vrn-B3*	*vrn-B3*	201	98.1	Jimai 13; Beijing 841; Nongda 311
	*Vrn-B3a*	3	1.46	Xiaobaimai; Pingyang 181; Lovin 10
	*Vrn-B3b*	1	0.49	Yanda 1817
	*Ppd-D1a*	184	89.8	Jinmai 50, Lumai 19, Yanzhan 4110
*Ppd-D1*	*Ppd-D1b*	21	10.2	Nongda 311, Beijing 6, Jinan 4
	2089-bp deletion in exon 8	193	94.1	Jinmai 50, Lumai 19, Yanzhan 4110
	TE insertion in intron 1	17	8.29	Lumai 14, Nongda 311, Yanda 1817
	5-bp insertion in exon 7	8	3.90	Beijing 6, Jinan 4, Pinchun 16

Up to 19 allelic combinations of vernalization response genes were discovered in terms of the *Vrn-A1, Vrn-B1, Vrn-D1*, and *Vrn-B3* loci in the wheat cultivars (Table 3). Distribution of different allelic combinations of vernalization genes suggested that the recessive allelic combination *vrn-A1/vrn-B1/vrn-D1/vrn-B3* was predominant, and three allelic combinations (*vrn-A1/vrn-B1/Vrn-D1a/vrn-B3, vrn-A1/vrn-B1/Vrn-D1b/vrn-B3*, and *vrn-A1/Vrn-B1a/vrn-D1/vrn-B3*) were also prevalent in Chinese winter wheat cultivars. Association of four above-mentioned allelic combinations of vernalization genes with heading and flowering times showed that cultivars with *vrn-A1/vrn-B1/vrn-D1/vrn-B3* possessed the latest heading and flowering times across 3 years, and cultivars with *vrn-A1/vrn-B1/Vrn-D1a/vrn-B3* possessed relatively earlier heading and flowering times than cultivars with *vrn-A1/vrn-B1/Vrn-D1b/vrn-B3* (Table 3).

**Table 3:** **The distribution of allelic combinations of vernalization genes in the Chinese bread wheat cultivars surveyed**.

**Allelic combination**	**Sample No**.	**Frequency (%)**	**2011–2012**		**2012–2013**		**2013–2014**	
			**HD**	**FD**	**HD**	**FD**	**HD**	**FD**
*vrn-A1/vrn-B1/vrn-D1/vrn-B3*	90	43.90	196.8	201.7	192.5	200.1	184.3	190.3
*vrn-A1/vrn-B1/Vrn-D1a/vrn-B3*	51	24.88	193.2	198.5	189.6	195.9	180.9	186.8
*vrn-A1/vrn-B1/Vrn-D1b/vrn-B3*	19	9.27	195.0	200.2	191.1	198.2	182.3	187.9
*vrn-A1/Vrn-B1a/vrn-D1/vrn-B3*	14	6.83	193.9	199.4	189.5	196.9	182.1	188.6
*vrn-A1/vrn-B1/Vrn-D1c/vrn-B3*	3	1.46	194.0	200.0	190.0	198.7	182.0	189.3
*vrn-A1/Vrn-B1b/vrn-D1/vrn-B3*	6	2.93	192.3	198.7	189.8	198.0	180.2	186.8
*vrn-A1/Vrn-B1a/Vrn-D1a/vrn-B3*	6	2.93	193.5	200.3	190.7	195.5	181.0	187.3
*Vrn-A1a/Vrn-B1a/vrn-D1/vrn-B3*	3	1.46	195.4	199.6	191.4	198.6	182.6	188.0
*Vrn-A1a/vrn-B1/vrn-D1/vrn-B3*	2	0.98	195.5	200.0	192.0	199.5	183.0	188.5
*vrn-A1/Vrn-B1b/Vrn-D1a/vrn-B3*	2	0.98	189.5	194.5	186.5	193.0	178.0	183.0
*vrn-A1/vrn-B1/vrn-D1/Vrn-B3a*	1	0.49	206	210	205	211	194	206
*vrn-A1/vrn-B1/vrn-D1/Vrn-B3b*	1	0.49	207	210	205	213	194	206
*Vrn-A1b/vrn-B1/vrn-D1/vrn-B3*	1	0.49	207	210	208	212	194	207
*Vrn-A1b/vrn-B1/Vrn-D1b/vrn-B3*	1	0.49	195	200	191	199	181	185
*vrn-A1/Vrn-B1a/Vrn-D1b/vrn-B3*	1	0.49	190	198	188	192	186	193
*vrn-A1/Vrn-B1b/Vrn-D1b/vrn-B3*	1	0.49	190	197	189	196	182	187
*vrn-A1/vrn-B1/Vrn-D1a/Vrn-B3a*	1	0.49	193	198	190	198	179	187
*vrn-A1/vrn-B1/Vrn-D1b/Vrn-B3a*	1	0.49	203	206	200	204	184	190
*vrn-A1/vrn-B1/vrn-D1_Null/vrn-B3*	1	0.49	194	198	189	197	183	189

### Distribution of Ppd-D1 gene in chinese winter wheat cultivars

A series of molecular markers (Ppd-P1–Ppd-P7; Supplemental Table 1) was used to identify the polymorphisms of the *Ppd-D1* gene as described by Guo et al. (2010). Three polymorphisms were found in bread wheat cultivars, namely, a 2089-bp deletion in exon 8, an TE insertion in intron 1, and a 5-bp deletion in exon 7. Two polymorphisms were absent, namely, a 16-bp insertion in exon 8 and a 24-bp plus a 15-bp insertion in the 2-kb upstream region. Distribution of polymorphisms of the *Ppd-D1* gene (Table 2) indicated that *Ppd-D1a* with the percentage of 89.8% and a 2089-bp deletion in exon 8 with the percentage of 94.1% were the most two popular genotypes in cultivars from the Yellow and Huai wheat region (detailed in Supplemental Data Sheet 1).

Furthermore, six combinations (Table 4) of the three *Ppd-D1* polymorphisms mentioned above were examined. Of the 205 cultivars surveyed, 184, 2, and 9 contained *Ppd-D1_Hapl-I* (presence of 5-bp insertion in exon 7 and absence of others), *Ppd-D1_Hapl-II* (presence of 5-bp insertion in exon 7 and 2089-bp in exon 8, and absence of others), and *Ppd-D1_Hapl-III* (presence of transposable element in intron 1, 5-bp insertion in exon 7, 2089-bp in exon 8, and absence of others), respectively. Two cultivars contained *Ppd-D1_Hapl-VIII* (absence of all the five polymorphisms) previously named by Chen et al. (2013a). Moreover, two new combinations of *Ppd-D1* polymorphisms were identified in this study and designated as *Hapl_IX* (presence of 2089-bp in exon 8 and TE in intron 1 and absence of others) in seven cultivars and *Ppd-D1_Hapl-X* (presence of TE in intron 1, and absence of others) in cultivar Beijing 6 in accordance with the nomenclature of Guo et al. (2010) and Chen et al. (2013a). Four known *Ppd-D1* haplotypes (*Ppd-D1_Hapl-IV*–*Ppd-D1_Hapl-VII*) were absent in these cultivars. The results suggested that *Ppd-D1_Hapl-I* was the most prevalent (90.24%) among the six combinations of *Ppd-D1* polymorphisms (Table 5).

**Table 4:** ***Ppd-D1* haplotypes identified in the Chinese bread wheat cultivars surveyed**.

**Haplotype**	**Sample no.**	**24 bp + 15 bp**	**2089 bp**	**TE**	**5 bp**	**16 bp**
*Ppd−D1_Hapl−I*	184	−	−	−	+	−
*Ppd−D1_Hapl−II*	2	−	+	−	+	−
*Ppd−D1_Hapl−III*	9	−	+	+	+	−
*Ppd−D1_Hapl−VIII*	2	−	−	−	−	−
*Ppd−D1_Hapl−V*	0	−	+	−	+	+
*Ppd−D1_Hapl−VI*	0	+	+	−	+	+
*Ppd−D1_Hapl−VII*	0	−	−	+	+	−
*Ppd−D1_Hapl−IX*	7	−	+	+	−	−
*Ppd−D1_Hapl−X*	1	−	−	+	−	−

*Insertions and deletions are indicated by + and −, respectively*.

**Table 5:** ***Ppd-B1* haplotypes identified in the Chinese bread wheat cultivars surveyed**.

**Haplotype**	**Sample no**.	**425 bp**	**994 bp**	**223 bp**	**2011–2012**		**2012–2013**		**2013–2014**	
					**HD**	**FD**	**HD**	**FD**	**HD**	**FD**
*Ppd-B1_Hapl-I*	89	No	No	No	195.9	201.0	192.3	199.3	183.8	189.8
*Ppd-B1_Hapl-II*	52	No	No	Yes	194.6	199.9	190.8	198.2	182.4	188.6
*Ppd-B1_Hapl-III*	13	Yes	No	No	194.7	199.8	190.8	197.5	182.5	188.5
*Ppd-B1_Hapl-IV*	2	Yes	No	Yes	199.0	203.5	193.5	202.0	185.0	190.5
*Ppd-B1_Hapl-V*	40	Yes	Yes	No	194.3	199.8	190.4	197.4	181.8	187.9
*Ppd-B1_Hapl-VI*	4	Yes	Yes	Yes	191.7	197.7	187.0	192.3	179.5	184.0
*Ppd-B1_Hapl-VII*	4	No	Yes	No	198.5	203.3	193.3	201.3	183.8	190.0
*Ppd-B1_Hapl-VIII*	1	No	Yes	Yes	194.0	200.0	192.0	199.0	182.0	188.0

### Copy number variations in *Ppd-B1* and *Vrn-A1* loci

The CNV is an important component of genomic diversity that has been proven to play a significant role in wheat adaptation (Diaz et al., 2012). In this study, three primer sets were used to identify the CNV of *Ppd-B1* gene in Chinese winter wheat cultivars following the method of Diaz et al. (2012); the Ppd-P8F/R (an expected 425-bp band for the gene/transposon junction in the truncated *Ppd-B1* copy of cultivar Chinese Spring), Ppd-P9F/R (an expected 994-bp band for the junction between intact *Ppd-B1* gene copies in Chinese Spring), and Ppd-P10F/R (an expected 223-bp band for the junction between intact *Ppd-B1* gene copies in cultivars Sonora64/Timstein/C591) were all identified (Supplemental Table 1). Identification results showed that the 425-, 994-, and 223-bp bands were successfully amplified in 59, 49, and 59 cultivars, respectively. Up to 89 cultivars without any bands when amplified with the abovementioned primer sets were identified. Subsequently, eight combinations were generated and designated as *Ppd-B1_Hapl-I*–*Ppd-B1_Hapl-VIII* (detailed in Table 5). Among these combinations, *Ppd-B1_Hapl-I* is the most popular genotype (43.4%). *Ppd-B1_Hapl-II* and *Ppd-B1_Hapl-V* are also prevalent genotypes (25.4 and 19.5%, respectively). Percentages of the remaining genotypes were <7% in the Chinese wheat cultivars surveyed (*Ppd-B1_Hapl-III* with 6.3%, *Ppd-B1_Hapl-IV* with 0.98%, *Ppd-B1_Hapl-VI* with 1.96%, *Ppd-B1_Hapl-VII* with 1.96%, and *Ppd-B1_Hapl-VIII* with 0.49%). Furthermore, four *Ppd-B1* genotypes with sample numbers of >10 (*Ppd-B1_Hapl-I, Ppd-B1_Hapl-II, Ppd-B1_Hapl-III*, and *Ppd-B1_Hapl-V*) were associated with heading time and flowering days. The results indicated that the cultivars with *Ppd-B1_Hapl-VI* (simultaneous presence of the 425-, 994-, and 223-bp bands) showed the shortest heading (179 days) and flowering days (184 days), whereas the cultivars with *Ppd-B1_Hapl-I* (simultaneous absence of the 425-, 994-, and 223-bp bands) demonstrated the longest heading time (184 days) and flowering time (190 days) under the condition of vernalization treatment (**Supplemental Table 2**).

Sequencing fragments amplified with the primer set Vrn_P16F/R (Supplemental Table 1) were used to identify the CNV of the *Vrn-A1* gene in this study. As described by Eagles et al. (2011), cultivars with single nucleotide C at +10,429 bp (relative to ATG) in exon 4 of *Vrn-A1* gene contained one copy of *Vrn-A1* gene and cultivars with double nucleotides C plus T at this locus contained two copies of *Vrn-A1* gene. Sequencing results showed that 47 wheat cultivars (22.9%) contained one copy of *Vrn-A1* gene and the remaining 158 cultivars (77.1%) contained two copies of *Vrn-A1* gene (**Supplemental Table 2**). These results suggested that the CNV of *Vrn-A1* gene was very prevalent in winter wheat cultivars from the Yellow and Huai valley of China.

## Discussion

The Yellow and Huai valley is the most important wheat region in China, accounting for 60–70% of the total harvested area and total wheat production of China. Backbone parents play an important role in increasing wheat yield as precious germplasms in wheat breeding programs. These precious germplasms are mainly composed of currently popular cultivars, historical cultivars, and landraces, as well as introduced cultivars from other countries or regions (e.g., International Maize and Wheat Improvement Center, USA, Europe, Australia, Canada, etc.). Chinese landrace Yanda 1817 is one of the most prevalent backbone parents, and the cross combination of Yanda 1817 and Triumph was regarded as the basis of the wheat breeding program of the Northern wheat region of China owing to their early maturity, good resistance, high yield, and strong adaptability (Zhuang, 2003; Han et al., 2009). Since the 1960s, 53 cultivars have been derived from Yanda 1817, including Nongda 183, Huabei 187, and Shijiazhuang 407. In the current study, we found that Yanda 1817 (194 DH, 206 DF) contained the rare allele *Vrn-B3b*, which possibly contributed to the relatively strong adaptability of this cultivar. Therefore, *Vrn-B3b* genotype may be fully considered as one of the relatively superior genotypes for breeding wheat cultivars with wide adaptability in bread wheat cultivars from the Yellow and Huai valley. Some introduced cultivars also played very important roles as core parents and contained many derivatives in Chinese wheat breeding programs (e.g., Lovin 10 from Romania, Nanda 2419, Funo and Abbondanza from Italy, Orofen from Chile, etc.). Molecular characterization of vernalization and photoperiod response genes for these core parents can provide useful information to further utilize the wheat germplasms in view of their adaptability and maturity in Chinese wheat breeding programs.

Previous studies indicated that increased expression of the *Vrn-D1* gene contributes to early flowering and maturity (Yan et al., 2004a; Chen and Dubcovsky, 2012). The dominant *Vrn-D1a* allele was found to result in early heading in a large set of Australian wheat genotypes (Eagles et al., 2010; Cane et al., 2013). Fu et al. (2005) suggested that the promoter and intron 1 regulatory sequences both affected the vernalization response, and mutations in the regulatory sequences reduced the expression of this gene. Zhang et al. (2012) indicated that the *Vrn-D1b* allele, with a single-nucleotide mutation at the promoter region, accelerated the heading and flowering times. However, the plants with *Vrn-D1b* homozygous allele headed 32 days later than the plants with *Vrn-D1a* homozygous allele without vernalization. In the present study, we found that cultivars with the dominant *Vrn-D1* allele headed earlier by an average of 3 days than cultivars with the recessive *vrn-D1* allele, which is consistent with the reports of Zhang et al. (2012) and Wang et al. (2015). Moreover, cultivars with *Vrn-D1a* allele headed and flowered ≈ 2 days earlier than cultivars with *Vrn-D1b* with vernalization. Cultivars with the novel allele *Vrn-D1c* showed significantly earlier heading and flowering times than cultivars with the recessive allele *vrn-D1* under non-vernalization treatment. This phenotype was ascribed to a 174-bp insertion contributing to higher expression level of *Vrn-D1* gene. However, these cultivars showed prolonged heading to flowering times under vernalization treatment. These results can provide useful information to further understand the molecular and genetic bases of vernalization in bread wheat. These results possibly suggested that *Vrn-D1c* genotype is relatively preferable among known *Vrn-D1* alleles in view of early maturity in bread wheat cultivars from the Yellow and Huai valley.

In the Yellow and Huai wheat region, low temperature is one of the important reasons to reduce wheat production. Cultivars with frost resistance were usually preferable for wheat breeders due to low temperature exposure of wheat plants for more than 3 months in this wheat region. Predominance of the recessive allelic combination *vrn-A1/vrn-B1/vrn-D1/vrn-B3* in bread wheat cultivars from the Yellow and Huai wheat region are possibly resulted from association of vernalization genes with frost resistance (Galiba et al., 1995; Zhu et al., 2014). Wheat plants with the recessive allelic combination *vrn-A1/vrn-B1/vrn-D1/vrn-B3* possibly possessed relatively stronger frost resistance than others (unpublished data), which possibly caused strong selection for this genotype in wheat breeding program in the Yellow and Huai wheat region.

To date, CNV has been recognized as a common type of polymorphism in the genomes of humans, animals, and plants (Żmieñko et al., 2014). CNVs of vernalization and photoperiod response genes significantly influence wheat flowering and maturity (Diaz et al., 2012). Cultivars with *Ppd-B1_Hapl-VI* demonstrated the earliest heading and flowering times in three cropping seasons among cultivars with eight different *Ppd-B1* polymorphisms. These results suggested that these cultivars (Yanshi 93(13)-1-1-0-1-1, R25, Shaan 89150, and Yunong 205), as well as cultivars with *Vrn-D1c* (Yunong 876, Shannong 418363, and Xinmai 18^*^) were useful wheat germplasms to develop relatively superior cultivars with early flowering and maturity. Diaz et al. (2012) showed that an increased copy number of *Vrn-A1* was strongly correlated with later flowering. In the current study, cultivars with two copies of *Vrn-A1* headed and flowered 1.4 days earlier than cultivars with only one copy of *Vrn-A1*. Diaz et al. (2012) also proved that a higher copy number of the *Ppd-B1* gene is responsible for photoperiod insensitivity. In the current study, wheat with the “Sonora64” allele showed flowering times slightly later than wheat with the “Chinese Spring” alleles. Moreover, the cultivars with both “Chinese Spring” allele and “Sonora64” allele headed the earliest among other cultivars. Cane et al. (2013) found the zero copy genotype, which is a new type of CNV. We also indentified this genotype with a high ratio (43.41%). Overall, we identified eight combinations of these three alleles. However, several new haplotypes remain unpublished. Therefore, further systematic research on CNV should be conducted.

### Conflict of interest statement

The authors declare that the research was conducted in the absence of any commercial or financial relationships that could be construed as a potential conflict of interest.

## Supplementary material

Supplemental Table 1 **PCR Primers for detecting vernalization response and photoperiod genes in the Chinese bread wheat cultivars**.

Supplemental Data Sheet 1 **Molecular characterization of vernalization and photoperiod response genes in all wheat cultivars surveyed from the Yellow and Huai valley of China**.
