# Supplementary material for: Allelic variation at the vernalization and photoperiod sensitivity loci in Chinese winter wheat cultivars (Triticum aestivum L.)
Source: Front Plant Sci. 2015 Jul 1;6:470. doi: 10.3389/fpls.2015.00470 (PMC4486769; doi:10.3389/fpls.2015.00470)
Supplement: Supplemental Table 1 — PCR Primers for detecting vernalization response and photoperiod genes in the Chinese bread wheat cultivars. [file Table1.DOCX]

Supplemental Table PCR Primers for detecting vernalization response and *Ppd-D1* alleles in bread wheat

| Name | Allele or haplotype | Primer | Targeted fragment (bp) | Annealing Temp. (°C) | Reference |
| --- | --- | --- | --- | --- | --- |
| Vrn_P1 | *vrn-A1 / Vrn-A1a / Vrn-A1b / Vrn-A1c* | F:GAAAGGAAAAATTCTGCTCG  R:TGCACCTTCCC(C/G)CGCCCCAT | 950+876 or 714 or 734 | 50 | Yan et al.  2004 |
| Vrn_P2 | *Vrn-A1b* | F:CCTGCCGGAATCCTCGTTTT  R:CTACGCCCCTACCCTCCAACA | 147  or 167 | 63 | Chen et al.  2013r |
| Vrn-P3 | *Vrn-A1c* | F:AGCCTCCACGGTTTGAAAGTAA  R:AAGTAAGACAACACGAATGTGAGA | 1170 | 65 | Fu et al.  2005 |
| Vrn-P4 | *vrn-A1* | F:GCACTCCTAACCCACTAACC  R:TCATCCATCATCAAGGCAAA | 1068 | 59 | Fu et al.  2005 |
| Vrn-P5 | *Vrn-B1a* | F:CAAGTGGAACGGTTAGGACA  R:CTCATGCCAAAAATTGAAGATGA | 709 | 63 | Fu et al.  2005 |
| Vrn-P6 | *vrn-B1* | F:CAAGTGGAACGGTTAGGACA  R:CAAATGAAAAGGAATGAGAGCA | 1149 | 58 | Fu et al.  2005 |
| Vrn-P7 | *Vrn-B1b* | F:CCAATCTCACATGCCTCCAA  R:ATGCGCCATGAACAACAAAG | 215  or 252 | 59 | Chen et al.  2013 |
| Vrn-P8 | *Vrn-D1a* | F:GTTGTCTGCCTCATCAAATCC  R:GGTCACTGGTGGTCTGTGC | 1671 | 63 | Fu et al.  2005 |
| Vrn-P9 | *vrn-D1* | F:GTTGTCTGCCTCATCAAATCC  R:AAATGAAAAGGAACGGAGCG | 997 | 59 | Fu et al.  2005 |
| Vrn-P10 | *Vrn-D1b or Vrn-D1c* | F:CGACCCGGGCGGCACGAGTG  R:AGGATGGCCAGGCCAAAACG | 612 bp or 787bp | 60 | Zhang et al.  2012 |
| Vrn-P11 | *Vrn-D1b* | F:CGACCCGGGCGGCACGAGTG  R:AGGATGGCCAGGCCAAAACT | 612 bp | 60 | Zhang et al.  2012 |
| Vrn-P12 | *vrn-B3* | F:ATGCTTTCGCTTGCCATCC  R:CTATCCCTACCGGCCATTAG | 1140  or 2030 | 56 | Yan et al. 2004 |
| Vrn-P13 | *Vrn-B3a* | F:CATAATGCCAAGCCGGTGAGTAC  R:ATGTCTGCCAATTAGCTAGC | 12000 | 59 | Yan et al.  2004 |
| Vrn-P14 | *Vrn-B3*c | F:GCTTTGAACTCCAAGGAGAA  R:ATAATCAGCAGGTGAACCAG | 1401 | 52 | Chen et al.  2013 |
| Vrn-P15 | *vrn-B3 /Vrn-B3* | F:ACTCATCATCACCACTTCCT  R:TAATGCTTAATTCGTGGCTG | 1499 | 51 | Chen et al.  2013r |
| Vrn-P16 | CNV of *Vrn-A1* | F:CATTGTTCCTTCCTGTCCCACCC  R:ATTACTCGTACAGCCATCTCAGCC | 1431 | 63 | Eagles et al.  2011 |
| Vrn-P17 | *Vrn-D1-RT* | F:CACCAAGGGAAAGCTCTACGAG  R:TCTCAACCTTCGCCTTCAGTTT | 163 | 60 | In this paper |
| Vrn-P18 | *β*-actin | F:GTTCCAATCTATGAGGGATACACGC  R:GAACCTCCACTGAGAACAACATTACC | 422 | 56 | Chen et al.  2013 |
| Ppd-P1 | *Ppd-D1a* | F:ACGCCTCCCACTACACTG  R:CACTGGTGGTAGCTGAGATT | 288  or 2377 | 54 | Beales et al.  2007 |
| Ppd-P2 | *Ppd-D1b* | F:ACGCCTCCCACTACACTG  R:GTTGGTTCAAACAGAGAGC | 414 or 453 | 54 | Beales et al.  2007 |
| Ppd-P3 | 16 bp insertion Exon 8 | F:GATGAACATGAAACGGG  R:GTCTAAATAGTAGGTACTAGG | 320 or 336 | 52 | Beales et al.  2007 |
| Ppd-P4 | TE deletion | F:AGGTCCTTACTCATACTCAATCTCA  R:CTCCCATTGTTGGTGTTGTTA | 2612 | 50 | Guo et al.  2010 |
| Ppd-P5 | 2kb deletion or TE absent | F:CCATTCGAGGAGACGATTCAT  R:CTGAGAAAGAACAGAGTCAA | 1005 | 55 | Guo et al.  2010 |
| Ppd-P6 | 5bp deletion Exon 7 | F:GAATGGCTTCTCCTGGTC  R:GATGGGCGAAACCTTATT | 1032 or 1027 | 50 | Guo et al.  2010 |
| Ppd-P7 | 5bp deletion Exon 7 | F:GTGTCCTTTGCGAATCCTT  R:TTGGAGCCTTGCTTCATCT | 184 or 179 | 53 | Guo et al.  2010 |
| Ppd-P8 | Truncated Ppd-B1 gene in the ‘Chinese Spring’ allele | F:TAACTGCTCCTCACAAGTGC  R:CCGGAACCTGAGGATCATC | 425 | 56 | Diaz et al.  2012 |
| Ppd-P9 | Intact Ppd-B1 copies in the ‘Chinese Spring’ allele | F:AAAACATTATGCATATAGCTTGTGTC  R:CAGACATGGACTCGGAACAC | 994 | 58 | Diaz et al.  2012 |
| Ppd-P10 | Intact Ppd-B1 copies in the ‘Sonora64’/‘Timstein’ allele | F:CCAGGCGAGTGATTTACACA  R:GGGCACGTTAACACACCTTT | 223 | 58 | Diaz et al.  2012 |
